# Dr. Joseph Taylor

**Published:** 1873-07

**Authors:** J. Taylor

**Affiliations:** Cincinnati


### DR. JOSEPH TAYLOR.

Cincinnati, June 15th, 1873.

DR. J. LESLIE—Dear Sir: In reply to your kind enquiry with reference to my brother, Dr. Joseph Taylor, recently deceased, I would state that he was born in September, 1806, Bainbridge, Ross Co., Ohio, and consequently he was in his sixty-seventh year when he died. The few of his early associates, professional and others, will recollect him in his youth as remarkable for his activity and athletic power; being full of life and vivacity he was ever a leading spirit in all innocent amusement. In 1830 I was pursuing the study or medicine under the instruction of Dr. John Harris, brothef of Prof. C. A. Harris, the latter practicing medicine in Greenfield, and the former in Bainbridge, Ohio. Dr. John Harris and myself were devoting more or less time to the practice of dentistry, and our success in this specialty led Dr. C. A. Harris as well as my brother to turn their attention to the same.

My brother of course had frequent opportunity of seeing Dr. Harris and myself operate, and without any consultation with any one determined to try his hand at dentistry. He set to work without our knowledge, made and secured such instruments as he conceived would be necessary, and started out on a trip through the interior of the state. He soon found his suavity of manner and genial address made it not difficult to secure business, but he also learned that he knew but little about dentistry. This short experience however fixed his purpose to take hold of dentistry as a profession. So that at once he put himself under a course of instruction and pursued it with much zeal and energy, so that in a few months he returned to the same place of his first adventure, and finished up operations he felt unabled to do at first.

This incident of his early life was characteristic of his whole professional carreer—he was quick to see his own defects, and was persevering to remedy them. This led him from year to year to extend his studies, and ultimately secured for him from one who knew him well the degree of Doctor f Dental Surgery—we mean Dr. C. A. Harris, of Baltimore.

After spending sometime in Ohio, West Virginia and Pennsylvania, he determined to visit the South, and in the winter of 1831, he located himself in Vicksburgh, Miss. This city was then first commencing that rapid growth, which gave it great notoriety, and afforded him a fine field for practice. His success was commensurate with his energy, and for five or six years this place was his regular winter abode, spending generally about six months of each year in this place.

In 1837 he married Miss Bell, of Greenfield, Ohio, and this led him to seek a more permanent abode, and hence a year or two thereafter he moved to Maysville, Ky. At this place Flemingsburg, and Mayslick, he found full employment, and only left at my urgent request to join me in the city.

Feb. 1850 he came to Cincinnati, and remained with me in practice until 1859, when some bronchial trouble admonished him that some other avocation which would expose him more to out-door exercise would be better for his health. For this purpose he engaged in the nursery and horticultural business, and through which his health became very much improved.

The disease which took him off was peri-pneumonia. His vigorous constitution made two or three efforts to throw off the disease, but the unfavorable weather of the spring kept renewing the difficulty until nature sank under the disease.

His most marked and stricking professional characteristic was his admirable tact in adopting means and appliances to meet difficulties in practice. This was positively the case in mechanical dentistry, and showed itself strongly in methods for correcting irregularity and for constructing obturators and palates.

When in Maysville, a gentlemen applied to him who had a large portion of the lower jaw and the palate completely destroyed by a gun shot wound ; he was utterly disfigured and unable to articulate a word understandingly, he made an artificial jaw, teeth and palate, and not only restored his speech but his good looks.

He was the first to extract the anterior superior molars by an outward and downward motion on a line with the direction of the palatine fang—securing their removal by the one effort or motion. In the removal of these teeth I think he excelled any one I ever saw. He had great patience and perseverence in any difficult operation if he thought success possible.

He took great interest in the early organization of our dental societies, and in discussion showed clear conception and much good practical sense. He had a high appreciation of dental science, and always aimed to elevate it.

He was not much given to authorship, but his first article on the use of letheon, in the first volume of the REGISTER, shows that his mind at once laid hold of the subject, and that he appreciated fully the constitutional idiosyncrasies, giving varied results in its administration. This and one or two articles on other subjects led me to think that a little practice would have made him more than ordinarily successful as a writer. His clear conception of a case, and his ability to express his views logically and forcibly justify this conclusion. He was one of the early pioneers of our profession in the West, and did his full share in placing it on that firm basis which it now enjoys. Of the many who rushed into dentistry during the period of short study from 1825 to the establishment of our dental colleges—say Baltimore and Ohio—but very few lived to accomplish more.

He was one of the few who then entered the profession who felt its importance and tried to meet its responsibilities aright.

One of the few who kept pace with its progress for thirty years, and only left it to seek a more healthy avocation.

J. TAYLOR.

---

It may seem as though perfection were reached in the various professions; but the old men who now totter along with the rapid present, thought so too, when they began, years ago.

